# Protein Interactome of Amyloid-β as a Therapeutic Target

**DOI:** 10.3390/ph16020312

**Published:** 2023-02-16

**Authors:** Vladimir F. Lazarev, Elizaveta A. Dutysheva, Igor E. Kanunikov, Irina V. Guzhova, Boris A. Margulis

**Affiliations:** 1 Institute of Cytology of the Russian Academy of Sciences, 194064 Saint Petersburg, Russia; 2 Biological Faculty, St. Petersburg State University, 199034 Saint Petersburg, Russia

**Keywords:** Alzheimer’s disease, amyloid fibrils, chaperones, GAPDH, protein–protein interaction, α-synuclein, tau-protein, therapeutical agent, cytotoxicity

## Abstract

The amyloid concept of Alzheimer’s disease (AD) assumes the β-amyloid peptide (Aβ) as the main pathogenic factor, which injures neural and other brain cells, causing their malfunction and death. Although Aβ has been documented to exert its cytotoxic effect in a solitary manner, there is much evidence to claim that its toxicity can be modulated by other proteins. The list of such Aβ co-factors or interactors includes tau, APOE, transthyretin, and others. These molecules interact with the peptide and affect the ability of Aβ to form oligomers or aggregates, modulating its toxicity. Thus, the list of potential substances able to reduce the harmful effects of the peptide should include ones that can prevent the pathogenic interactions by specifically binding Aβ and/or its partners. In the present review, we discuss the data on Aβ-based complexes in AD pathogenesis and on the compounds directly targeting Aβ or the destructors of its complexes with other polypeptides.

## 1. Introduction

**Figure 1:**
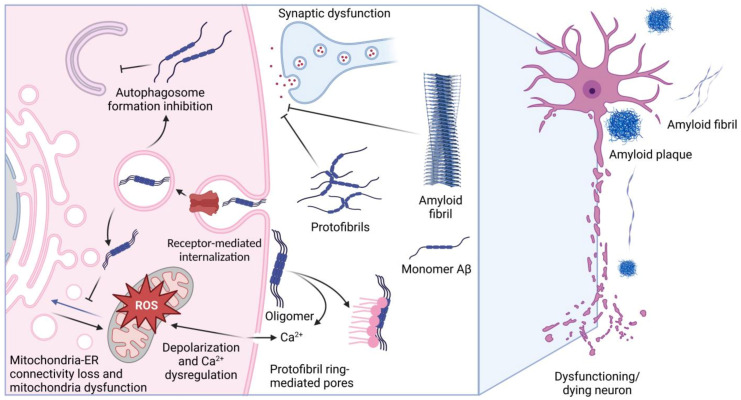
Targets of Aβ pathogenicity. The figure schematically shows the main known mechanisms of the pathogenic action of Aβ and amyloid fibrils, which cause dysfunction of neurons and their death. The figure was created with BioRender.com, accessed on 28 December 2022.

Alzheimer’s disease (AD) and similar pathologies stemming from cytotoxic polypeptide species cover a wide range of knowledge and methods, from the molecular organization of a single cell to the behavioral reactions of the whole organism. Although it is hard to imagine, thousands of papers, monographs, and doctoral theses support the idea that only a few species of amyloid precursor protein (APP) demonstrate pathogenic effects when outside or inside a neural cell [1]. The amyloid hypothesis has a firmly established basis, and the meaningfulness of APP-derived peptides as pathogenic triggers seems to have dominated in the last few decades [2,3,4]. APP peptides, such as Aβ1–42 or Aβ1–40, occurring intracellularly or exported in excess by brain cells, may form oligomers, small aggregates, or fibrils and damage neurons by inducing oxidative stress, suppressing the function of membrane channels, or affecting transport/sorting mechanisms (Figure 1). Some Aβ molecules are localized to the structures called senile plaques, which, according to some data, may have cytotoxic activity [3]. Notably, the amount of Aβ accumulating in the extracellular matrix, cerebrospinal fluid, and blood may indicate the progress of AD. Gradually, such techniques are used in prostheses for diagnosing AD [5,6].

The physical properties and biological activity of Aβ depend on its amino acid composition and on its post-translational modifications, features that confine the aggregation ability and toxicity of the peptides [7]. As a solitary factor, Aβ can attack neurons, damaging their synapses or other structures and causing a pathologic chain of events including the disruption of intercellular contacts; examples of such deleterious effects come from investigations performed on glial and microglial cells [8]. It is also noteworthy that Aβ was found to bind some low-molecular-weight compounds, metabolites, and metals that are also able to modulate the pathogenicity of the peptide [9,10]. Lastly, several proteins were found to specifically bind Aβ, and their activity may be modulated by the peptide, or vice versa, the peptide’s activity may be affected by the interactors. These phenomena form a complex picture of the Aβ-based effect on brain tissue, as shown in Figure 1. In this review, we present the data on Aβ-binding proteins and on the effects of such complexes in the context of AD pathology. It seems that the role of such interactions is underestimated in modern neurobiology, while it is likely that it is precisely such interprotein bonds that determine the course of disease pathogenesis. In the present review, special attention is paid to small molecules able to affect the above interactions and, therefore, to be employed for AD therapy.

## 2. Aβ and Its Protein Interactors

### 2.1. Structural Features of Aβ

Aβ is the proteolytic fragment of APP protein, the product of the APP gene, localized to the 21st chromosome [11]. Having several isoforms, this transmembrane protein interacts with heparan sulfate, type I collagen, fibronectin, and other components of the cell matrix, making APP an important player in the regulation of cell adhesion. In addition, APP is involved in neuronal development, particularly neurite growth and differentiation [12]. It was shown that APP plays an important role in excitatory as well as inhibitory synaptic transmission. In particular, APP interacts with glutamatergic [13], cholinergic [14], and gamma-aminobutyric acid B receptor (GABAB) [15]. APP was found to participate in the regulation of Ca^2+^ homeostasis by interacting with NMDA receptors and VGC channels [16].

Amyloid precursor protein has a long N-terminal extracellular domain, a smaller C-terminal cytoplasmic domain, and a single-span transmembrane domain [17]. There are two paths of APP processing, where non-pathogenic fragment 1–40 is produced by the consecutive cleavage of α- and γ-secretases, and the amyloidogenic 1–42 fragment of precursor protein is processed by β- and γ-secretases [9]. The resulting Aβ structure has a sequence of 12 aliphatic amino acids at the C-terminus and two hydrophobic stretches, one of which is situated C-terminally and the second lies in the interim of 12–25 residues. Furthermore, the peptide has several sites of interaction with metal ions [18].

In the normal brain, secreted amyloids, primarily in monomeric form, participate in synaptic transmission, protection against oxidative stress, and signaling [19,20]. In conditions when the concentration of Aβ increases up to 10^−7^ M, it can form oligomers and demonstrate neurotoxicity [11]. Having a sequence of five positively charged amino acids at the N-terminus, Aβ can interact with negatively charged cell surface phospholipids, such as sphingomyelin and phosphoethanolamine, and integrate into the membrane [9]. It was also demonstrated that binding with Aβ triggers the internalization of NMDA receptors and facilitates Aβ penetration [21]. Moreover, Aβ impairs mitochondrial function [22] by increasing the contact between sub-areas of mitochondria and ER membranes, thereby causing mitochondrial dysfunction and alterations in autophagosome assembly [23].

The amyloid peptide structure defines its ability to self-assemble; monomers are considered non-toxic, but so are oligomers. Some authors use the term “smallest neurotoxic species” to refer to soluble Aβ oligomers. Many previous investigations confirm this point of view; for example, Shankar et al. showed that the injection of Aβ oligomers collected directly from the brains of AD patients into rat hippocampus impairs synaptic plasticity and causes memory deterioration [24]. It was shown that Aβ oligomers have an even higher ability to be incorporated into lipid membrane than their monomeric form, and they may form protofibril rings, which are able to create pores within a plasma membrane. This causes ion influx and the disruption of Ca^2+^ homeostasis, followed by mitochondrial disfunction and neuronal death [25].

The assembly of Aβ into higher-order molecular forms proceeds by the mechanism of nucleated polymerization. This mechanism suggests that nucleation components, including the extracellular matrix, lipid surfaces, such as plasma membranes, and metal ions serve as the “scaffold” for different forms of Aβ [18]. Protofibrils demonstrate high neurotoxicity; e.g., in a transgenic model of AD, many protofibrils were detected, and the progressing neuronal dysfunction appeared before the formation of fibrils and plaques [26]. In addition, Yasumoto et al. reported that protofibrils induce reactive oxygen species generation, lipid peroxidation, leading to calcium dysregulation, and the depolarization of iPSC-generated human cortical neuron plasma membrane [27]. Contrary to protofibrils, the fibrils and plaques formed by Aβ may not exert neurotoxicity [28], but many data prove the causative role of the latter in synaptic dysfunction [3].

Similar to other cellular polypeptides, Aβ undergoes post-translational modifications, including phosphorylation, nitrosylation, pyroglutamination, glycosylation, etc.

For example, Aβ phosphorylation at Ser8 is traditionally associated with an increase in the ability of the peptide to form aggregates, as well as an increase in its toxic effect [29]. Interestingly, the interaction of phosphorylated Aβ with zinc has the opposite effect; the formation of stable dimers in the presence of zinc prevents aggregation and toxicity of the peptide [30]. Moreover, it was shown that administration of the phosphorylated Aβ to B6C3-Tg transgenic mice prevented the formation of amyloid plaques in the hippocampus of the animals [31]. Likewise, the isomerization of Asp7 was shown to induce Aβ toxicity. When studying the response of cellular models of AD to Aβ with isoAsp7, the latter was found to be more toxic than its naïve form and to induce apoptosis [32,33], while in the case of intact Aβ peptides, necrosis dominated [34]. Another well-known modification of Aβ—pyroglutamate—increases the hydrophobicity of the peptide, prevents its degradation, and increases the tendency to form aggregates and toxicity [35]. It was also shown in transgenic mice of the TBA2 (expresses Aβ with N-terminal glutamine) line that this modification of Aβ accelerates the progression of AD in vivo [36]. Indeed, there are clearance mechanisms that withstand the toxicity of Aβ oligomers, including the glymphatic system, perivascular drainage, and blood–brain barrier, but their function is often impaired in the aged brain [5,37]. Another way of amyloid removal that is associated with its internalization, packing into endosomes, and lysosomal degradation can also exhibit negative alterations with aging. Impaired lysosomal function in older brain tissue may cause the local condensation of amyloids into the lumen of late endosomes, forming cytotoxic structures in brain cells; this compartment becomes a suitable site for Aβ to aggregate [38]. It can be assumed that Aβ is released from endosomes into the cytoplasm, proving that the intracellular location of the peptide may provide a cytotoxic effect [39].

Thus, the structural (including isomerization, oligomerization, and aggregation) and chemical (including phosphorylation) modifications of Aβ can greatly affect its toxic properties. Moreover, such changes in toxicity may depend on Aβ partner proteins.

### 2.2. Protein Interactome of Aβ

Various forms of Aβ are neurotoxic by themselves; however, in many cases, amyloid-mediated pathogenicity is due to its effects on the plasma membrane, synaptic space, cytosol, endosomes, etc., where Aβ interacts with a great number of cellular proteins, some of which are implicated in cell damage.

The proteomic analysis of amyloid plaques shows approximately 2000 proteins with significant variation in samples of AD patients with a distinct clinical story. Liao et al. found that the level of at least 26 proteins is higher in plaques compared with healthy brain samples. Among these proteins are tau, proteins involved in vesicular transport proteolysis (cathepsin D, lysosomal ATPase, ubiquitin-activating enzyme E1) and inflammation (GFAP and vimentin), and HSP90 chaperone [40]. Data from the proteomic analysis of brain- and CSF-derived extracellular vesicles from AD patients show that heat shock protein 70 (HSP70), puromycin-sensitive aminopeptidase (NPEPPS), glyceraldehyde-3-phosphate dehydrogenase (GAPDH), transthyretin, cystatin C, and prostaglandin F2 receptor negative regulator (PTGFRN) can also be implicated in AD pathogenesis, since their expression varies during the pathogenic process [41]. Many of the above proteins found to be involved in the progression of Alzheimer’s disease are potential interactors of the amyloid (Table 1). The list of Aβ interactors including K(lysine) acetyltransferase 5 (Tip60) [42], fibulin 1 [43], solute carrier family 25 (mitochondrial carrier; adenine nucleotide translocator), member 4 (SLC25A4) [44], and essential meiotic structure-specific endonuclease 1 (EME1) [45] as the most abundant, according to the Biogrid database, are provided below (Table 1).

Taking into consideration the data presented above, there are plenty of Aβ partner proteins, and some of them have attracted the attention of investigators in the search for new targets of anti-AD therapy.

### 2.3. Proteins Whose Function Is Affected by Aβ

During the progression of AD in the intercellular space, as well as in the cytoplasm of brain cells, a significant accumulation of the Aβ peptide is observed. Accordingly, greater amounts of the peptide interactors could be recruited to impact the pathogenic function of the peptide. Conversely, in conditions of enhanced protein–protein interaction probability, Aβ itself may affect the function of its binders, and in following chapters we discuss the outcomes of such interactions.

#### 2.3.1. Tau-Protein

Microtubule-associated protein tau (MAPT, tau) is one of Aβ’s best known partners. The normal function of tau is to regulate the formation of tubulin microtubules, predominantly in neuronal axons [48] and, to a lesser extent, in dendritic cells [49]. Other functions of tau have been discovered in the past decade, including translational regulation, insulin signaling, and others [50]. In AD pathogenesis, tau is known to form protofibrils, which convert into fibrillar cytotoxic structures, and the process can occur both in the cerebrospinal fluid and inside neural cells [51].

**Figure 2:**
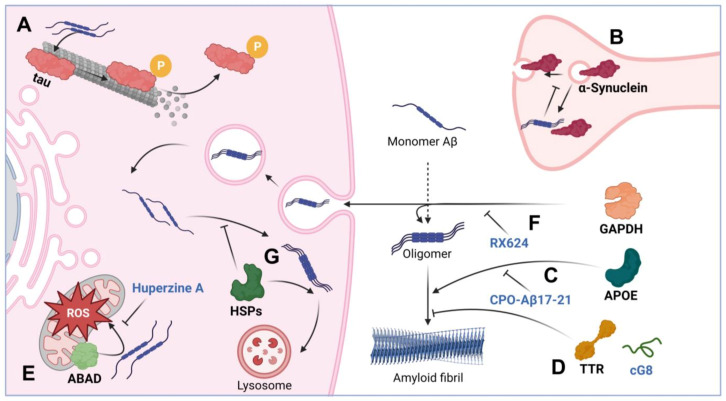
Modulation of Aβ toxicity with interactor proteins and their dissociators. The figure schematically shows the interaction: (**A**) Aβ with tau causing microtubule disruption. (**B**) Aβ interaction with α-synuclein causing vesicle trafficking disorder. (**C**) APOE promotes the formation of amyloid fibrils; CPO-Aβ17–21 peptide may block this interaction. (**D**) TTR and its cG8 mimetic prevent the formation of toxic amyloid fibrils. (**E**) ABAD interacts with Aβ, activates ROS in mitochondria; this process can be prevented with the help of Huperzine A. (**F**) GAPDH activates the formation of amyloid fibrils due to direct interaction with Aβ and promotes horizontal transfer of the amyloid; the hydrocortisone derivative RX624 is able to block the pathogenic action of GAPDH. (**G**) Chaperones prevent the formation of toxic amyloid fibrils and are involved in the lysosomal and proteolytic degradation of Aβ. The figure was created with BioRender.com, accessed on 28 December 2022.

It is believed that Aβ causes the hyperphosphorylation of tau, which, on the one hand, disrupts the normal function of the latter, and, on the other hand, leads to fibrillation and neurotoxicity [52]. Notably, tau phosphorylation is an ordinary event in cell physiology [53,54], and eight possible phosphorylation sites have been discovered on all six isoforms of tau [55,56]. The mechanisms of Aβ’s effect on tau hyperphosphorylation are not yet clear, despite the fact that there is much evidence of the two polypeptides’ coaggregation in the brains of AD patients [57,58]. In a recent report, the effect of Aβ on the kinases phosphorylating tau was evidenced [59], while earlier data suggest the possibility of targeting by Aβ available tau phosphorylation sites [60] (Figure 2A).

#### 2.3.2. α-Synuclein

α-synuclein is a neuronal protein which is thought to be involved in the regulation of synaptic transmission through vesicle trafficking [61]. During the progression of AD, and in other neurodegenerative pathologies, including Parkinson’s disease, Aβ and synuclein may interact to increase each other’s amyloidogenic potential [62]. It has recently been shown in vitro and in vivo in Tg2576 transgenic mice that Aβ can serve as a trigger for α-synuclein aggregation [63]. It was also established that Aβ is able to influence α-synuclein functioning via phosphorylation at Ser129, which has been proven in vitro in SH-SY5Y cells and in brain tissue homogenates [64]; the enhanced phosphorylation corroborated with the formation of insoluble aggregates of α-synuclein [65], and a violation of its synaptic function [66,67] (Figure 2B). Interestingly, the formation of synuclein aggregates (Lewy bodies) is associated with the indications of synucleopathies, thus suggesting that the effect of Aβ on α-synuclein in some way blurs the line between Alzheimer’s and Parkinson’s disease. However, the exact mechanism of the effect of Aβ on α-synuclein phosphorylation is still elusive; as a possible option, death-associated protein kinase 1 is worth mentioning, for which the ability to phosphorylate α-synuclein at Ser129 with subsequent synuclein aggregation has also been demonstrated [65,68].

Thus, the interaction of Aβ with normal proteins and peptides often leads to a violation of the physiological functions of the latter, and even, in some cases, to their involvement in cytotoxic complexes.

### 2.4. Proteins Affecting Aβ Toxicity

The cytotoxicity of Aβ1–42, being the basis of its pathogenicity, can be mediated through a variety of mechanisms based on polypeptides, whose role and interactions with Aβ have been proven (Figure 2); some of these proteins are considered in the following chapter.

#### 2.4.1. Apolipoprotein E

Apolipoprotein E (APOE) is a protein with a well-established role in AD pathogenesis. Normally, it is an extracellular protein, whose major function is the transport of cholesterol and lipids between neuronal and glial cells [69]. According to the generally accepted view, isoform 4 of the APOE protein is associated with a high risk of rapid progression of AD, while isoform 2, on the contrary, is associated with a low risk [70]. The interaction between APOE and Aβ is traditionally considered in two aspects: (i) in the pro-survival context of Aβ clearance [71,72], it has been repeatedly demonstrated that APOE binding Aβ ensures its transport across the blood–brain barrier [73], as well as its uptake and utilization by cellular proteolytic systems [74]; and (ii) in the pro-aggregation context, APOE may induce Aβ oligomerization and aggregation (Figure 2C). Thus, the interaction of extracellular APOE with Aβ in a number of experiments initiated the formation of toxic aggregates based on Aβ [75,76]. Interestingly, the opposite statement is also correct—the interaction of Aβ with APOE causes the formation of neurotoxic oligomers based on APOE—which was convincingly demonstrated in SK-N-SH cells [77].

#### 2.4.2. Transthyretin

Transthyretin is a protein necessary for the transport of thyroxine and retinol; its concentration is highest in the cerebrospinal fluid, where its share can be up to 20% of the total protein content [78]. Mutations in the gene encoding transthyretin are commonly associated with amyloidosis [79]. Transthyretin was first described as an Aβ-binding protein in 1994 [80]. Probably due to its ability to sequester Aβ, transthyretin may decrease the amplitude of the protopathic stress induced by amyloids, which has been repeatedly demonstrated, including in APPswe/PS1A246E transgenic mice [81]. The results of experiments in vivo and in AD patients indicate that, being in the blood plasma or in the cerebrospinal fluid, transthyretin is able to prevent the formation of Aβ-based amyloid fibrils (Figure 2D) [82]. The protective mechanism of transthyretin is associated with its ability to directly bind Aβ and thus prevent the processes of primary and secondary nucleation [83]. This action reduces not only the toxicity of amyloid-based fibrils, but also their ability to grow further.

#### 2.4.3. ABAD

Another protein that has been shown to influence Aβ-mediated cytotoxicity is Aβ-binding alcohol dehydrogenase (ABAD). ABAD is a mitochondrial protein that can bind Aβ predominantly in the mitochondria. In AD pathogenesis, the level of ABAD expression is increased, primarily in the hippocampus and cerebral cortex [84]. We can consider the relationship between the functioning of mitochondria and the progression of AD to be convincing and repeatedly proven [85]. Mitochondrial dysfunction is known to contribute to a reduction in energy metabolism, the elevation of free radical formation, and the violation of cellular calcium homeostasis, all being relevant to the pathogenesis of AD [86]. One of the possible mechanisms of mitochondrial dysfunction in AD is associated with the accumulation of Aβ in the mitochondria; in particular, in mitochondrial cristae [87]. At the same time, many researchers agree that the accumulation of the pathogenic peptide in mitochondria occurs through its anchoring with ABAD [88] (Figure 2E). Since the Aβ–ABAD interaction is believed to promote Aβ-mediated mitochondrial and neuronal dysfunction, this complex can become a therapeutic target in the treatment of AD [89].

#### 2.4.4. GAPDH

Glyceraldehyde-3-phosphate dehydrogenase (GAPDH), another protein essential for normal cell function, is known as one of the basic enzymes driving glycolysis; however, it is also implied in numerous crucial events in cell physiology, particularly in response to oxidative stress [90]. GAPDH can be covalently crosslinked with Aβ [91,92,93] and can significantly increase the toxicity of its complex with Aβ occurring in the intercellular space (Figure 2F) [94]. Using the method of enzyme immunoassay, we established that tissue transglutaminase (tTG) enhances the interaction between GAPDH and Aβ1–42; moreover, tTG accelerated the formation of co-aggregates of GAPDH and Aβ1–42 by covalent crosslinking through the 15th glutamine on the Aβ molecule and lysines on the GAPDH molecule, [95]. According to atomic force microscopy data, GAPDH with Aβ in the presence of tTG may form amyloid-like structures. Notably, the level of GAPDH expression correlated with AD progression in a chemically induced AD model in rats—a high level of GAPDH expression led to severe memory dysfunction and the formation of intensive amyloid plaques. Finally, we found that in patients with AD, the GAPDH–Aβ complex is present in the cerebrospinal fluid, and the amount of the complex correlates well with the severity of disease [94]. These data suggest that GAPDH can be a target for AD therapy, which was proved in our work: the use of the GAPDH binder, a hydrocortisone derivative RX624, in the 5xFAD transgenic mice model led to a significant slowdown in the progress of memory impairment and the formation of amyloid plaques.

#### 2.4.5. Chaperones

Chaperones comprise a group of proteins that are able to bind Aβ and reduce the level of the peptide-initiated pathological processes (Figure 2G). Chaperones provide cell protection under stressful conditions, prevent the development of apoptosis, participate in the processes of protein folding and renaturation, and are necessary for the recognition and labeling of damaged proteins to be utilized through autophagy [96] and proteasomal machineries [97].

The ability of Hsp70 and Hsp90 to bind Aβ oligomers and prevent their further growth was demonstrated in vitro [98]. Moreover, the concentration of chaperones sufficient for the effective blocking of the processes of aggregation was 50 times lower than the concentration of Aβ, suggesting that the inhibitory activity is most effective in the early stages of oligomerization.

Recent data demonstrate that the interaction of the Hsp110 co-chaperone with Aβ can also prevent the formation of Aβ fibrils [99]. Using a Drosophila AD model, Yakubu et al. showed that the β-subunit of the Hsp110 substrate-binding domain is required to provide a protective interaction with amyloid.

Interestingly, the cognate form of the Hsp70 protein, Hsc70, is able to bind the amyloid precursor protein via the KFERQ-motif and ensure its utilization by the mechanism of chaperone-mediated autophagy [100]. It is believed that such utilization of the precursor reduces the potential toxicity of Aβ. Based on the mechanism of chaperone-mediated autophagy, a new approach for AD therapy has recently been proposed, based on labeling toxic Aβ oligomers with KFERQ-motifs, which ensured the utilization of Aβ by lysosomes [101].

In general, the ability of the chaperone machine to bind Aβ and prevent the formation of amyloid aggregates and their toxicity in vitro is well-established [102,103], but experimental confirmation of this hypothesis in vivo has not yet been presented.

Notably, the data obtained from the clinical analysis of the chaperones and their interactors, together called the epichaperome, led to the conclusion that the state of this united interactome is tightly associated with the pathogenesis of many neurological diseases, including AD [104]. In this case, the therapeutic effect can be obtained with the drug correction of the epichaperome—networks of interprotein intra- and extracellular interactions, including those involving chaperones and Aβ [105].

#### 2.4.6. Cystatin C

Cystatin C is a protein capable of forming amyloid structures on its own under certain circumstances [106,107,108]. Nevertheless, Selenica et al. recently established in vitro that the direct binding of cystatin to Aβ in a 1:1 molar ratio prevented the further formation of oligomers and aggregates of the latter [109]. Moreover, using transgenic mice with the overexpression of cystatin, it was found that increasing the concentration of cystatin C prevented the deposition and formation of Aβ-based amyloid plaques [110,111]. Later, using cultured primary hippocampal neurons, it was demonstrated that the addition of purified recombinant human cystatin C significantly reduced the cytotoxicity of growing Aβ oligomers [112]. Moreover, for a significant decrease in the toxicity of amyloid, near-physiological concentrations of cystatin C are sufficient. Despite such results, attempts to use cystatin C as a target for AD therapy have not yet been undertaken.

From the presented data, it follows that normal non-pathogenic proteins can affect the cytotoxic properties of Aβ in completely diverse ways (Figure 2). The complexes formed with the peptide in some cases have a higher toxicity compared with Aβ oligomers, as exemplified by GAPDH; sometimes, on the contrary, the interaction of proteins with Aβ significantly reduces the toxic potential of the complex (such as cystatin C).

## 3. Chemicals Targeting Aβ and Its Intermolecular Complexes

Aβ is one of the most ubiquitous drug targets in neurology, and to date, approximately 100 different substances remain in different stages of clinical trials; 28 of them were claimed in 2019 to target the Aβ or APP cleavage mechanism [ClinicalTrials.gov; Alzforum.org]. Generally, an efficient anti-amyloid drug must recognize and bind oligomeric and protofibrillar amyloid forms to prevent their conversion to cytotoxic fibrils (Table 2). As such, the most well-known anti-AD drug, aducanumab, is based on a monoclonal antibody generated against Aβ aggregates and humanized for therapeutic use. In the experiments performed on transgenic mice, the antibody was reported to ameliorate cognitive functions, to clean the brain tissue of the peptide, and to activate microglia phagocytosis [113]. However, the ability of aducanumab to recognize toxic species of Aβ is limited [114]; this and problems with the clinical use of the medicine have cast doubt on its future. Hence, the approval of aducanumab by the Food and Drug Administration in 2021 was one of the most criticized FDA decisions in recent years [115]. Very recently, Lecanemab, another humanized monoclonal antibody that binds to Aβ soluble protofibrils, demonstrated reduced markers of amyloid in persons with early AD in Phase III clinical trials while the authors assume that longer trials are warranted to estimate the effectiveness. It is likely that the new drug Lecanemab (also based on antibodies) will be more effective than aducanumab, but so far it has certain side effects [116].

The list of compounds able to reduce Aβ pathogenicity includes a number of synthetic or natural peptides, some of which share an amino acid sequence similar to the hydrophobic domains of Aβ (aa16–20, aa11–23, or aa32–37), and were reported to decrease the formation of fibrils consisting of the protein target [117]. An example of such compounds is the peptide-targeting amyloid fragment 13–23 designed using the molecular dynamics approach; the peptide was found to block amyloid–amyloid binding, the first step of the formation of toxic Aβ species [118]. More recently, Kim et al. presented a collection of synthetic peptides able to recognize various domains of Aβ molecules and to serve as diagnostic tools, if their clinical use is not excluded [119]. Overall, it is likely that compounds modifying or binding amyloid-β would influence its multiple interactions with other molecules.

Indeed, during the last decade, a few novel small molecules able to inhibit Aβ misfolding and enhance its clearance were reported, one of which, the LS-4 amphiphilic compound, demonstrated the extremely high binding affinity toward various Aβ forms, especially for soluble Aβ oligomers. In the 5xFAD mouse model, LS-4 reduced the amount of amyloid plaques and phosphorylated tau aggregates, the most-known manifestations of AD [120].

Proteins binding Aβ may impact its pathogenicity, and therefore their complexes with the polypeptide have become essential druggable targets for AD therapy [121]. One of the key Aβ-binding proteins is ApoE. Liu et al. found that the inhibition of its interaction with Aβ using CPO-Aβ17–21 peptide led to a decrease in cognitive impairment and neuroprotection in an AD APP/PS1 transgenic mouse model [122]. Recent studies have shown that blocking the ability of APOE to initiate Aβ oligomerization also has a therapeutic effect—a similar approach has been successfully tested in imipramine and olanzapine preparations on 5xFAD transgenic mice, TgF344-AD transgenic rats, and a primary neuronal culture obtained from these animals [123].

Transthyretin was also demonstrated to bind Aβ in cell and animal AD models; this binding leads to a reduction in the toxicity of Aβ-based fibrillar structures, and several approaches have been proposed to establish such an effect. First, it was shown that a cyclic peptide cG8 comprising the Aβ-binding domain of transthyretin reduced the deposits of fibrillar Aβ; the authors stated that success was achieved, and believed that the optimization of the mimetic should be continued [124]. A similar approach was used in another study, in which an Aβ-binding peptide fragment of transthyretin was able to prevent the toxic effect of Aβ on SH-SY5Y and PC-12 cells [125].

The ability of ABAD to bind Aβ was firmly attributed to AD pathogenesis, and a few attempts have been made to prevent the interaction between the two proteins. First, the prevention of the interaction between Aβ and ABAD in mAPP mice using a specific ABAD-decoy peptide protected neurons from Aβ-mediated toxicity [126]. Secondly, Huperzine A, an alkaloid isolated from lycopodium, was found to reduce the deposition of Aβ and the ABAD level, as well to weaken Aβ–ABAD interaction and finally to ameliorate cerebral mitochondrial function in APP/PS1 mice [127]. Evidence of the neuroprotective effect of ABAD blocking comes also from the recent data obtained in experiments on human SH-SY5Y cells and the culture of primary neurons of 5xFAD transgenic mice; ABAD inhibition was performed with allopurinol derivatives and resulted in a reduction in Aβ-induced mitochondrial dysfunction [128].

Another potential therapeutic approach is to block the formation of the GAPDH–Aβ complex, which was found to be extremely cytotoxic to neural cells. Thus, it was shown that the hydrocortisone derivative RX624, capable of binding GAPDH, in a 5xFAD transgenic mice model of Alzheimer’s disease, not only inhibited the formation of the GAPDH–Aβ complex, but also prevented memory impairment [94]. Additionally, an extensive panel of GAPDH inhibitors was presented that prevented the aggregation of oxidized protein and reduced its intra- and extracellular cytoxicity; the list includes deprenyl, PGL-135, N-phenoxyacetyl-L-cysteine [129], RX409, RX426, and RX648 [130]. It is believed that such compounds may be useful in preventing the formation of the GAPDH–Aβ complex in the context of AD pathogenesis.

Inducers of chaperone synthesis are worth mentioning as a separate therapeutic approach aimed at blocking the formation of Aβ complexes with other proteins [131]. Since the molecular chaperones, in particular Hsp70, act as strong factors dissociating protein complexes with the peptide by binding Aβ [98], the induction of the chaperone can impact the therapeutic development of a variety of neurodegenerative diseases, including AD. Among the inducers with demonstrated efficacy in in vitro models of Alzheimer’s disease are celastrol [132], geranylgeranylacetone [133], 17-AAG Hsp90 inhibitor [134,135], and the recently discovered PQ-29 and other pyrrolyl- and indolylazine derivatives [136,137].

**Table 2:** Therapeutical agents with action based on prevention of Aβ-containing pathological complexes formation.

Therapeutical Agent (Class of Agents)	Potential Function	Reference
Aducanumab (specific antibodies) and other agents preventing formation of Aβ fibrils formation	Prevention of Aβ assemblage into cytotoxic fibrils	[113]
Synthetic and natural peptides that may block amyloid–amyloid binding	Interaction conditioned by the similarity to the hydrophobic domains of Aβ	[117]
Small molecules able to inhibit Aβ misfolding and enhance its clearance (LS4, for example)	Specifically binding to different soluble forms of Aβ	[120]
CPO-Aβ17–21 peptide	Blocking the ability of APOE to initiate Aβ oligomerization	[122]
Cyclic peptide cG8	TTR-mimetic peptide comprising its Aβ-binding domain	[124]
Huperzine A and other ABAD blocking compounds	ABAD inhibition reduces Aβ-induced mitochondrial dysfunction	[127]
GAPDH–Aβ complex inhibitors	Blocking the formation of the GAPDH–Aβ complex and reduction of its cytotoxicity	[94,138]
Chaperone synthesis inducers	Newly synthesized chaperones block the formation of Aβ complexes with other proteins	[131,136,137]

## 4. Conclusions

Proteins interacting by any way with various forms of Aβ can become markers of AD and there are samples where they are employed so. Aβ was found to kill neurons or other cells by influencing function of a few of important proteins appearing in extracellular space and particularly concentrating in certain locations, such as in interstitial liquids. In such domains the concentration of the latter complexes may increase to values becoming very toxic and this may be a reason for neuronal death. We further hypothesize that the process of cell death once initiated would be expanded and result in the release of more Aβ interactors from dying cells, including alarmins and activation of microglia response, as established in many instances. This mortality circle can be interrupted by using molecules specifically dissociating Aβ complexes. To date, extensive information has been accumulated on possible dissociators of Aβ-containing complexes. In most cases, these are small molecules of completely different chemical nature. Probably, the modulation of the toxic activity of Aβ-containing aggregates with the participation of various normal proteins is a biologically significant process that plays a decisive role in the pathogenesis of Alzheimer’s disease. Thus, blocking the formation of numerous protein complexes involving Aβ may be a more effective therapeutic strategy than inhibiting the formation of oligomers and aggregates formed only with Aβ.

## Figures and Tables

**Table 1:** Potential interactors of Aβ and their function in AD progression.

Protein Name	Sample Source	Potential Function	Reference
Ubiquitin-activating enzyme E1	Plaques	Ubiquitin-dependent proteosomal degradation of Aβ	[40]
GFAP	Plaques	Activation of astrocytic immune response and astrocytic damage	[40]
HSP70	Extracellular vesicles	Involvement in ubiqitin-proteosomal and lysosomal degradation of Aβ, disaggregation of fibrils, and immune response activation	[41]
PTGFRN	Extracellular vesicles	Positive regulation of APP procession and Aβ production	[46]
GAPDH	Extracellular vesicles	Enhancement of Aβ aggregation and toxicity	[41]
TTR	Extracellular vesicles	Blockage of Aβ nucleation	[41]
CST3	Extracellular vesicles	Negative regulation of Aβ oligomers and aggregate formation	[41]
Tip60	Protein–protein interaction	In complex with HDAC2 protection against AD-associated pathologies	[47]
Fibulin 1	Protein–protein interaction	Regulation of APP cleavage	[43]
SLC25A4	Protein–protein interaction	Unknown	[44]
EME1	Protein–protein interaction	Unknown	[45]

## Data Availability

Not applicable.
